# Association of nutrition behavior and food intake with overweight and obesity among school-aged children and adolescents in Pakistan: a cross-sectional study

**DOI:** 10.3934/publichealth.2024040

**Published:** 2024-06-21

**Authors:** Moazzam Tanveer, Ejaz Asghar, Umar Tanveer, Nadeem Roy, Asifa Zeba, Sameer Badri Al-Mhanna, Xiaoran Ma, Alexios Batrakoulis

**Affiliations:** 1 School of Physical Education and Sport Training, Shanghai University of Sport, Shanghai 200438, China; 2 Department of Allied Health Sciences, Health Services Academy, Islamabad 44000, Pakistan; 3 Department of Mass Communication, University of Lahore, Lahore 54000, Pakistan; 4 School of Physical Education, Shanxi University, Taiyuan 030006, China; 5 Department of Education, International Islamic University, Islamabad 44000, Pakistan; 6 Department of Physiology, School of Medical Sciences, Universiti Sains Malaysia, Kubang Kerian 16150, Kelantan, Malaysia; 7 School of Physical Education, Xi'an Physical Education University, Shaanxi 710064, China; 8 Department of Physical Education and Sport Science, University of Thessaly, Trikala 42100, Greece

**Keywords:** overweight and obesity, body mass index, diet-related behaviors, dietary habits, children and adolescents

## Abstract

**Purpose:**

This study aimed to assess the association between nutrition behavior, food intake, being overweight, and obesity among school-aged children and adolescents aged 9 to 17 years. Additionally, it sought to examine how these factors influence being overweight and obese within this population.

**Methods:**

A population-based cross-sectional study was conducted with a representative multistage cluster sample of 4200 Pakistani school-aged children and adolescents aged 9 to 17 years from 62 schools across seven random districts in Punjab province, Pakistan. Underweight (BMI < 5th percentile), overweight (85th ≤ BMI < 95th percentile), and obese (95th percentile ≤ BMI) were defined using the US Center for Disease Control (CDC) 2000 criteria, and a Chi-square test utilized for comparison. The Pearson correlation coefficient (r) assessed any correlations, while a linear regression analysis explored the predictive power of Nutrition Behavior/Food Intake factors (independent variables) on body-weight (dependent variable). A logistic regression analysis estimated the simultaneous influence of multiple factors on the dichotomous outcomes, and the 95% confidence intervals (CI) were calculated. The statistical significance level was set at *p* < 0.05.

**Results:**

The study was comprised of 4108 Pakistani school children aged 9 to 17 years (mean age = 13.92 years, 59.3% boys) from 62 schools. Among them, the prevalence of being overweight and obese individuals was 19.4% and 10.7%, respectively. Factors such as skipping breakfast (OR 2.45, 95% CI 1.53–3.93, *p* < 0.001), consuming vegetables less than once a week (OR 4.12, 95% CI 3.06–5.55, *p* < 0.001), consuming soft drinks three or more times a week (OR 4.74, 95% CI 3.73–6.04, *p* < 0.001), and consuming fast food three or more times a week (OR 10.56, 95% CI 8.16–13.67, *p* < 0.001) were associated with a higher risk of obesity.

**Conclusion:**

Being overweight and obese pose significant concerns among school-aged children and adolescents in Pakistan, showing a troubling upward trend. Poor nutrition behaviors, including frequenting fast-food restaurants and low consumption of fruits and vegetables, contribute to these issues. It is imperative to comprehend these risk factors to formulate impactful policies and dietary interventions that target childhood obesity in Pakistan. Identifying vulnerable populations and implementing tailored intervention strategies are essential for public health efforts. While further interventions may be needed to reduce the body mass index (BMI) and manage being overweight and obese, the findings of this study provide valuable insights into addressing these critical health challenges.

## 1. Introduction

Children being overweight and obese represent persistent challenges among children and adolescents [1]. Early development of unhealthy behaviors can lead to children enduring negative health impacts [2]. Various external factors can influence children's health behaviors and outcomes [3]. In recent years, childhood and adolescent obesity have emerged as significant global public health concerns [4]. This epidemic has affected both high and low-income countries, with the prevalence of overweight and obese children doubling or even tripling since 1970 [5]. According to the World Health Organization (WHO), in 2016, 18% of children and adolescents aged 5–19 years were either overweight or obese globally, with notable variations in the obesity rates across regions [6]. Importantly, being overweight or obese has profound short and long-term repercussions on the physical, mental, and emotional health of children and their future as adults [1]. Examining the role of diet-related behaviors and habits in the development and prevention of being overweight and obese during childhood and adolescence is crucial, as these behaviors are established early, but can potentially be modified in the future, albeit with some difficulty. Moreover, addressing obesity and weight loss during adulthood becomes increasingly challenging, particularly after the age of 35 [7].

The cause of obesity is multifactorial, involving a long-term imbalance between energy expenditure [5],[6]. Various diet-related behaviors prevalent among adolescents, such as skipping breakfast, irregular family meals, dining at fast-food restaurants, and excessive screen time, along with dietary habits like consuming sugar-sweetened beverages, have been studied in their relation to obesity and being overweight [8]. Specifically, skipping breakfast has been linked to a higher body mass index (BMI) and an elevated risk of obesity or being overweight [5],[9]. While the frequent consumption of fast food has been associated with childhood obesity due to the promotion of unhealthy food choices, the findings have been inconsistent [10]. Moreover, although consuming a diverse range of vegetables and fruits is essential for a healthy diet, the association between vegetable and fruit intake and obesity or being overweight during childhood and adolescence remains inconclusive [11]. A recent review concluded that the available data do not strongly support a protective role of high fruit and vegetable intake in reducing the risk of childhood obesity [12].

Obesity is becoming increasingly prevalent worldwide [13]. Pakistan, which is classified as a low- and middle-income country [14], has a significant portion of its population aged between 0 to 19 years old, which is approximately 54% of the population [15] However, despite this youthful demographic, the country faces serious challenges related to hunger, ranking 92nd out of 116 countries in the global hunger index for 2021 [16]. Pakistan grapples with the dual burdens of overnutrition and poor nutrition, with approximately 50% of its population being either overweight or obese, placing it tenth among 188 countries [17]. Alarmingly, Pakistan has witnessed a steady rise in early fatalities linked to being overweight among both males and females over time [18]. Projections from the World Obesity Federation suggest that 5.4 million Pakistani school-aged children will be obese by 2030 [19]. Despite these concerning statistics, Pakistan has yet to implement operational policies to address being overweight, obesity, and physical inactivity, as noted in the WHO Diabetes country profiles [20]. Although research on obesity among school-aged children and adolescents in Pakistan is limited, there is a pressing need for baseline data to evaluate its prevalence [21]. Childhood obesity is a global epidemic, affecting an estimated ten percent of school-aged children worldwide, with a quarter of them classified as obese [22]. Given the significant health risks associated with childhood obesity and being overweight, alongside insulin resistance, hypertension, type 2 diabetes, and psychosocial issues, urgent prevention and control measures are imperative. The school environment offers a promising platform for interventions, allowing for the regulation and modification of physical activity, food choices, and attendance patterns to effectively combat childhood obesity [8]–[11],[47]–[49]. The health of children and adolescents relies on food intake that provides sufficient energy and nutrients to promote optimal physical, cognitive, and social growth and development. A balanced diet and regular physical activity are essential for proper growth, development, and maintaining good health [38]. Dietary behavior encompasses all phenomena related to food choices, eating habits, and dietary intake [39]–[41].

The aim of this study is to determine the prevalence of obesity and being overweight among adolescents aged 9 to 17 years old in Pakistan using a nationally representative sample, and to investigate its association with diet-related behaviors and habits. The ultimate objective is to provide evidence-based recommendations to prevent and manage obesity and being overweight in these age groups. The findings of this study have broader implications for the development of effective interventions, policies, and campaigns aimed at alleviating the burden of being overweight and obese among school-aged children and adolescents, not only in Pakistan, but also in other regions worldwide.

## 2. Materials and methods

### 2.1. Study design, and participants

In the summer of 2023, a population-based cross-sectional study was conducted among school-going children and adolescents aged 9 to 17 years in seven randomly selected districts of Punjab, Pakistan. A stratified multistage random cluster sampling method was employed to enroll a total of 4200 school-aged children and adolescents from 62 schools located in Lahore, Gujranwala, Gujrat, Sheikhupura, Narowal, Hafizabad, and Sialkot. Among the invited students, 4108 students (97.80%) completed useful questionnaires, while 92 students (2.2%) were disqualified due to insufficient information. Children aged 9–11 years old and adolescents aged 12–17 years old were sampled from the 4th to 12th grades, excluding grades 1 to 3 due to their inability to complete the study questionnaire [1]. Public schools were selected after obtaining permission from the Punjab school education department, which issued permission letters. Private school administrations independently granted permission upon visitation. The Punjab school education department (https://sis.punjab.gov.pk/) provided a list of schools from both urban and rural areas. A nominal fee of 20 PKR was charged in the public schools, while private schools were charged 10,000.00 PKR to cover any disparities in the participants socioeconomic status. In cases where a school refused to participate, another institution was randomly selected [3]. Additionally, the survey included participation from the education and Rescue-1122 departments, which volunteered to take part.

The Institutional Review Board of Shanghai University of Sport authorized the study protocol [1816111009], and permission to conduct the study was obtained from the teachers and principals of the participating schools. All children, adolescents, and their parents or guardians were informed that participation was completely voluntary.

### 2.2. Data on anthropometry and classification of body mass index

On pre-arranged dates, the sampled schools were visited by the Rescue-1122 professionals, who conducted anthropometric measurements of weight and height in the classroom. Data from the students were collected directly, and the confidentiality of the participants' responses were ensured throughout the process. The body weights and heights were measured first, with the weights being recorded to the nearest 0.1 kg and the heights to the nearest 0.5 cm [23]. Then, the BMIs were calculated by dividing the person's weight in kilograms by their height in meters squared (kg/m^2^). BMI classifications—underweight (BMI < 5th percentile), normal weight (5th percentile ≤ BMI < 85th percentile), overweight (85th percentile ≤ BMI < 95th percentile), and obese (95th percentile ≤ BMI)—were determined based on age and gender-specific BMI percentiles according to US Center for Disease Control (CDC) 2000 standards for children and adolescents aged 2 to 20 years [24],[25]. Trained rescue professionals conducted all measurements to ensure accuracy and consistency.

### 2.3. Diet-related behaviors and food consumption

We collected data on the dietary behavior of Pakistani school-aged children and adolescents using 7-day recall surveys. The questionnaire, which was originally written in English, was read aloud and translated to children in lower grades to ensure comprehension and included the following questions: 1. how often did you eat breakfast in the last seven days? (Reliability coefficient = 0.78); 2. how many times per day did you eat fruit, such as apples, bananas, oranges, or mangoes, in the previous 7 days? (Reliability coefficient = 0.78); 3. during the past 7 days, how many times per day did you usually eat vegetables, such as tomatoes, carrots, cabbage, spinach, salad, cauliflower, ladyfingers, or corn? (Reliability coefficient = 0.78), since according to WHO recommendations, eating at least 400 g, or five portions, of fruit and vegetables per day reduces the risk of NCDs and helps to ensure an adequate daily intake of dietary fiber: https://www.who.int/news-room/factsheets/detail/healthy-diet; 4. during the past 7 days, how many times per day did you usually drink carbonated soft drinks, such as Pepsi, Coca-Cola, 7Up, Sprite, Miranda, Fanta, Dew, or Big Apple? (Reliability coefficient = 0.78); and 5. During the past 7 days, on how many days did you eat food from a fast-food restaurant, such as McDonald's, Kentucky Fried Chicken (KFC), Pizza Hut, Subway, AFC, or Rahat? (Reliability coefficient = 0.78). We adopted these questions from the Global School-based Student Health Survey (GSHS) 2016 Pakistan GSHS Questionnaire (Available from: http://www.cdc.gov/gshs or http://www.who.int/chp/gshs/en).

### 2.4. Statistical analysis

The data was analyzed using an IBM SPSS v.26 Statistical Analysis. The BMI was categorized into underweight (BMI < 5th percentile), normal weight (5th percentile ≤ BMI < 85th percentile), overweight (85th percentile ≤ BMI < 95th percentile), and obese (95th percentile ≤ BMI), and was calculated based on the CDC US 2000 BMI chart for children and adolescents aged between 2 and 20 [25]. A frequency distribution analysis was conducted to determine the prevalence of the body-weight status in the present study. A bivariate analysis was used to compare the prevalence of the body-weight status (dependent variable) with Intrapersonal-Level Nutrition Behavior/Food Intake (independent variables) using a chi-square test as the trend test [26]. To measure the relationship between the dependent and independent variables, the Pearson correlation coefficient (r) was used to determine the degree of correlation between the independent variables and the body-weight dependent variable. A linear regression analysis was used to explore the predictive power of the nutrition behavior/food intake factors (independent variables) in relation to the body-weight (dependent variable). The simultaneous influence of numerous factors on the dichotomous outcome was estimated using a logistic regression analysis. The odds ratios (OR) with 95 % confidence intervals were calculated. The statistical significance was determined using *p* < 0.05.

## 3. Results

According to Table 1, the study included a total of 4,108 participants, with 59.3% being boys and 40.7% girls. The participants were divided into two age groups, with 18.6% being children aged 9 to 11 and the remaining as adolescents aged 12 to 17. The vast majority of the participants (96.2%) identified as Muslims, while 3.8% belonged to other religious groups. The sample represented both metropolitan (59.9%) and rural (40.1%) areas, indicating a diverse geographical representation. In terms of school types, 22.2% of the participants attended private schools, while 77.8% attended public schools.

**Table 1.** Demographic characteristics of Pakistani school-aged children and adolescents aged 9–17 years, descriptive statistics (n (%)).

Characteristics	Primary School	Middle School	Secondary School	Higher Secondary School	*p-value*
Sample size, n (%)	844 (20.5)	1580 (38.5)	1227 (29.9)	457 (11.1)	
Age (year, mean ± SD)	10.91 ± 1.23	13.15 ± 1.35	15.30 ± 1.13	16.82 ± 0.44	
Sex, n (%)					
Boy	463 (54.9)	902 (57.1)	816 (66.5)	256 (56.0)	<0.001
Girl	381 (45.1)	678 (42.9)	411 (33.5)	201 (44.0)	
Religion, n (%)					
Muslim	799 (94.7)	1513 (95.8)	1191 (97.1)	449 (98.2)	0.003
Non-Muslims	45 (5.3)	67 (4.2)	36 (2.9)	8 (1.8)	
Residence, n (%)					
Urban	656 (77.7)	869 (55.0)	759 (61.9)	176 (38.5)	<0.001
Rural	188 (22.3)	711 (45.0)	468 (38.1)	281 (61.5)	
School Type, n (%)					
Public	650 (77.0)	1196 (75.7)	902 (73.5)	446 (97.6)	<0.001
Private	194 (23.0)	384 (24.3)	325 (26.5)	11 (2.4)	
BMI (kg/m^2^, mean ± SD)	17.01 ± 3.35	19.06 ± 4.07	21.01 ± 4.70	22.41 ± 4.46	

Note: BMI: body mass index.

**Table 2.** Chi-square test to assess the overweight and obesity and its association with nutrition behavior factors: Sex-specific trend.

Characteristics	Sex	Body Weight-status				*χ^2^*	*p-value*
		Underweight*n (%)*	Healthy*n (%)*	Overweight*n (%)*	Obesity*n (%)*		
**Sex**							
Boys		444 (18.2)	1319 (54.1)	455 (18.7)	219 (9.0)	26.99	<0.001
Girls		247 (14.8)	860 (51.5)	342 (20.5)	222 (13.3)		
**Breakfast**							
Dose not eat	Boys	9 (20.9)	17 (39.5)	7 (16.3)	10 (23.3)	5.18	0.159
	Girls	12 (15.2)	34 (43.0)	20 (25.3)	13 (16.5)		
Usually eat	Boys	72 (17.6)	199 (48.5)	83 (20.2)	56 (13.7)	7.58	0.055
	Girls	73 (12.9)	273 (48.1)	120 (21.1)	102 (18.0)		
Always eat	Boys	363 (18.3)	1103 (55.6)	365 (18.4)	153 (7.7)	26.85	<0.001
	Girls	162 (15.8)	553 (54.0)	202 (19.7)	107 (10.4)		
**Fruits**							
<once/week	Boys	171 (16.4)	565 (54.2)	214 (20.5)	93 (8.9)	14.30	0.013
	Girls	78 (13.4)	302 (52.0)	115 (19.8)	86 (14.8)		
1–2 times/week	Boys	201 (19.7)	561 (54.9)	170 (16.7)	89 (8.7)	17.88	<0.001
	Girls	124 (15.9)	408 (52.2)	153 (19.6)	97 (12.4)		
≥3 times/week	Boys	72 (19.3)	193 (51.7)	71 (19.0)	37 (9.9)	25.74	<0.001
	Girls	45 (14.6)	150 (48.7)	74 (24.0)	39 (12.7)		
**Vegetables**							
<once/week	Boys	106 (16.6)	321 (50.2)	131 (20.5)	81 (12.7)	70.03	<0.001
	Girls	53 (7.8)	265 (39.1)	172 (25.4)	188 (27.7)		
1–2 times/week	Boys	218 (19.4)	627 (55.9)	193 (17.2)	84 (7.5)	11.60	0.009
	Girls	117 (17.3)	385 (56.8)	146 (21.5)	30 (4.4)		
≥3 times/week	Boys	120 (17.8)	371 (54.9)	131 (19.4)	54 (8.0)	45.50	<0.001
	Girls	77 (24.4)	210 (66.7)	24 (7.6)	4 (1.3)		
**Soft drinks**							
<once/week	Boys	279 (18.8)	857 (57.7)	279 (18.8)	71 (4.8)	53.79	<0.001
	Girls	133 (14.6)	497 (54.6)	163 (17.9)	117 (12.9)		
1–2 times/week	Boys	123 (20.0)	346 (56.2)	126 (20.5)	21 (3.4)	51.23	<0.001
	Girls	89 (15.5)	266 (46.3)	138 (24.0)	82 (14.3)		
≥3 times/week	Boys	42 (12.5)	116 (34.6)	50 (14.9)	127 (37.9)	39.63	<0.001
	Girls	25 (13.4)	97 (52.2)	41 (22.0)	23 (12.4)		
**Fast food**							
<once/week	Boys	318 (19.1)	947 (56.7)	312 (18.7)	92 (5.5)	31.99	<0.001
	Girls	187 (16.8)	637 (57.2)	214 (19.2)	76 (6.8)		
1–2 times/week	Boys	96 (18.3)	298 (56.8)	84 (16.0)	47 (9.0)	11.36	0.010
	Girls	51 (14.2)	175 (48.7)	79 (22.0)	54 (15.0)		
≥3 times/week	Boys	30 (12.3)	74 (30.5)	59 (24.3)	80 (32.9)	14.16	0.003
	Girls	9 (4.5)	48 (24.2)	49 (24.7)	92 (46.5)		
**Total**		691 (16.8)	2179 (53.0)	797 (19.4)	441 (10.7)		

Based on the latest data from Table 2, the prevalence of being overweight was 19.4%, with 10.7% of participants being classified as obese. When comparing the nutrition behavior factors with sex-specific prevalence of weight status, some notable trends emerge. For instance, among boys, the prevalence of skipping breakfast was higher in those who were obese (23.3%) compared to girls (16.5%). Similarly, among overweight individuals, a higher percentage of girls (25%) did not eat breakfast compared to boys (16.3%). However, the association between skipping breakfast and a weight category was not statistically significant. Additionally, while the majority of girls who were overweight and obese reported usually eating breakfast (21.1% and 18.0%, respectively), no significant association was found between breakfast consumption and a weight category. Furthermore, among girls, a higher percentage of overweight (24.0%) and obese (12.7%) individuals reported eating fruits three or more times a week, whereas boys had lower percentages (15.9% overweight and 7.5% obese) when consuming fruits at a similar frequency. Moreover, girls exhibited a higher prevalence of being overweight and obese compared to boys when considering vegetable consumption. Soft drink and fast-food consumption patterns also varied by sex and weight category, with notable differences observed between boys and girls.

The findings indicate that there are sex-specific differences in the prevalence of a weight status and its association with intrapersonal level nutrition behavior factors. Girls generally had higher rates of being overweight and obese compared to boys. The majority of the nutrition behavior factors were significantly associated with a weight status, except for skipping breakfast (*p* = 0.159) and usual breakfast (*p* = 0.055) consumption, which did not show any significant associations.

**Table 3.** Correlation between nutrition behavior and body weight-status.

Characteristics		1	2	3	4	5	6
1	Body Weight-status	—					
2	Breakfast	−0.101**	—				
3	Eat fruits	−0.012	0.042**	—			
4	Eat vegetables	−0.202**	0.139**	0.161**	—		
5	Drink soft drinks	0.148**	−0.069**	0.216**	0.125**	—	
6	Fast-food	0.260**	−0.082**	0.095**	−0.010	0.260**	—

Note: N = 4108; **p < 0.01.

Based on the data presented in Table 3, several bivariate correlations between body weight status and various nutritional behavior and food intake parameters were evident. Skipping breakfast showed a negative relationship with weight status (*r* = −0.101**), indicating that those who skipped breakfast may be more prone to a higher body weight-status. Similarly, a weak negative correlation exists between weight status and fruit consumption (*r* = −0.012), suggesting that an increased fruit intake might be linked to a lower weight status, albeit not strongly. Conversely, a negative correlation is observed between weight status and vegetable consumption (*r* = −0.202**), indicating that an increased vegetable consumption may relate to a lower weight status. Conversely, a positive correlation is noted between weight status and carbonated soft drink consumption (*r* = 0.148**), suggesting that an increased intake of carbonated drinks may be associated with a higher weight status. Lastly, a robust positive correlation is found between weight status and fast-food consumption (*r* = 0.260**), indicating that individuals that consume more fast-food are likely to have a higher body-weight status.

**Table 4.** Analysis of linear regression nutrition behavior and body weight-status.

		Unstandardized Coefficients		Standardized Coefficients	
Characteristics		B	SE	*β*	*t*	Sig.
	Constant	2.317	0.082		28.121	<0.001
1	Breakfast	−0.074	0.025	−0.045	−3.011	0.003
2	Fruits	−0.029	0.018	−0.024	−1.597	0.110
3	Vegetables	−0.235	0.017	−0.205	−13.601	<0.001
4	Soft drinks	0.141	0.019	0.117	7.504	<0.001
5	Fast food	0.286	0.019	0.226	14.883	<0.001

Note: SE = Standard error.

According to Table 4, which presents estimates from the linear regression analysis, the influence of nutrition-related parameters on body weight status is evident. Breakfast consumption has a significantly negative influence on weight status (*β* = −0.04, SE = 0.02, *p* = 0.003), suggesting that individuals who regularly consume breakfast are more likely to have a lower weight status. Similarly, vegetable consumption exhibits a significantly negative influence on weight status (*β* = −0.20, SE = −0.23, *p* < 0.001), indicating that an increased vegetable intake is associated with a lower weight status. Conversely, soft drink consumption shows a significantly positive influence on weight status (*β* = 0.11, SE = 0.06, *p* < 0.001), implying that an increased soft drink consumption correlates with a higher weight status. Likewise, fast food consumption demonstrates a significantly positive influence on weight status (*β* = 0.22, SE = 0.28, *p* < 0.001), indicating that an increased fast-food intake is linked to a higher weight status. However, fruit consumption does not significantly influence the weight status (*β* = −0.02, SE = 0.02, *p* = 0.110). The overall analysis yields an *R*^2^ value of 0.122, indicating that the included nutrition-related parameters explain approximately 12.2% of the variance in the weight status. The F-test result (*F* (5, 4102) = 114.216, *p* < 0.001) suggests that the regression model is statistically significant.

Based on the outcomes presented in Table 5, two binary logistic regressions were conducted to assess the association between the nutrition behavior factors and being overweight and obese. For being overweight, significant predictors included having a vegetable consumption frequency less than once a week and once or twice a week, having a soft drink consumption frequency once or twice a week, and having a fast-food consumption frequency three or more times a week, with odds ratios ranging from OR 1.25 to OR 1.61 (*p* < 0.01). However, skipping breakfast and consuming fruits less than once a week did not emerge as significant predictors. Regarding obesity, significant predictors included skipping breakfast, usually eating breakfast, having a vegetable consumption frequency less than once a week, having a soft drink consumption frequency three or more times a week, and a fast-food consumption frequency less than once a week and three or more times a week, with odds ratios ranging from OR 2.03 to OR 9.95 (*p* < 0.001). Conversely, consuming fruits less than once a week, consuming vegetables once or twice a week, and consuming soft drinks once or twice a week were not significant predictors of obesity.

**Table 5.** Odds ratios from two logistic regression analyses of nutrition behavior and risk factors associated with overweight and obesity.

Characteristics	Overweight vs. Non-overweight	Obese vs. Non-obese
	Unadjusted OR (95% CI)	Unadjusted OR (95% CI)
**Breakfast**		
Dose not eat	1.22 (0.79–1.89)	2.45 (1.53–3.93)***
Usually eat	1.12 (0.94–1.34)	2.03 (1.64–2.51)***
Always eat	Ref.	Ref.
**Fruits**		
<once/week	0.93 (0.75–1.17)	0.98 (0.74–1.31)
1–2 times/week	0.80 (0.64–1.00)	0.91 (0.69–1.21)
≥3 times/week	Ref.	Ref.
**Vegetables**		
<once/week	1.61 (1.30–1.99)***	4.12 (3.06–5.55)***
1–2 times/week	1.25 (1.16–1.54)*	0.78 (0.76–1.50)
≥3 times/week	Ref.	Ref.
**Soft drinks**		
<once/week	Ref.	Ref.
1–2 times/week	1.25 (1.06–1.49)**	1.11 (0.86–1.42)
≥3 times/week	0.93 (0.73–1.200)	4.74 (3.73–6.04)***
**Fast food**		
<once/week	Ref.	Ref.
1–2 times/week	0.97 (0.79–1.17)	2.00 (1.54–2.60)***
≥3 times/week	1.39 (1.09–1.76)**	9.95 (7.77–12.74)***

Note: Level of significance **p < 0.01, ***p < 0.001, CI = Confidence Interval, OR = Odds Ratio; Reference category (respectively): Ref.

## 4. Discussion

According to current estimates, 19.4% (18.7% of boys and 20.5% of girls) of Pakistani school-aged children and adolescents were overweight and 10.7% (9.0% of boys and 13.3% of girls) were obese. Another study conducted in Lahore among primary school children found that 17% of children aged 5 to 12 were overweight and 7.5% were obese [27]. Additionally, a study from the Hyderabad urban region in 2013 found that 12% of students in grades 6 to 10 were obese and 8% were overweight [28]. Another study conducted in Karachi among school children aged 11 to 15 years old found that 19.1% of the children were overweight and 10.8% were obese [21]. A local survey conducted in Lahore found that 11.9% of students in private schools in grades 6 and 7 were obese, while 21.8% were overweight [29]. In 2018, another local study was conducted on children aged 3 to 18 years in Multan, and the results showed that 10% of the students were overweight and 5% were obese [30]. According to the World Obesity Federation estimated in 2018, 6.6% of Pakistani children were obese and 10.7% were overweight [31].

In the current study, girls were found to have a higher likelihood of being overweight and obese. This finding is consistent with research conducted by Cai et al. among Chinese school children aged 9 to 17 years, where 14.4% of children and adolescents were overweight and 11.9% were obese [1]. Conversely, boys in China appeared to be less affected than girls [1],[2]. Similarly, a study by Ogden et al. among US adolescents reported overweight rates of 15.5% and 15.3% among adolescents and children, respectively, which aligns with the findings of the current study [32]. Furthermore, research by Czy et al. found that girls were more likely to be underweight, emphasizing the need for interventions aimed at reducing the risk of being overweight and obese among children in specific regions, such as the Lower Silesia region of Poland [35]. These varying results underscore the importance of considering geographical and cultural diversity when studying BMI correlations, suggesting that each population should be individually examined before generalizing universal theories. Given the diverse findings, Pakistan, with its unique geographical and cultural characteristics, exhibited a range of body-weight correlations.

Among the diet-related behaviors examined in the current study, skipping breakfast was not found to be significantly associated with a weight status, whereas breakfast consumption was negatively associated with BMI and exhibited a high significant odds ratio with obesity. This finding contrasts with previous studies where skipping breakfast was positively associated with being overweight, particularly among girls. Substantial evidence suggests that breakfast consumption is negatively associated with being overweight [9]. Children who skipped breakfast were more likely to be overweight and obese compared to those who regularly consumed breakfast. Gender-specific trends in skipping breakfast and the prevalence of being overweight revealed a significant association among girls but not boys [3]. Skipping breakfast has been consistently associated with being overweight/obese in schoolchildren and adolescents [8].

In this study, significant associations were observed between weight statuses using the chi-square test, with particularly high odds ratios associated with vegetable consumption and obesity. However, no significant associations were found between fruit and vegetable consumption and being overweight in the current study. These findings are consistent with several previous studies [3],[33]. A recent review by Newby on the role of plant-based diets and foods in preventing obesity concluded that the available data on the specific role of fruits and vegetables were either inconsistent or generally inconclusive, often due to methodological limitations [34]. Nevertheless, the lack of a consistent association with being overweight and obese does not negate the importance of consuming a variety of fruits and vegetables daily, given their known beneficial effects in preventing major chronic diseases such as cardiovascular diseases and certain cancers.

In this study, a significant association was observed between consuming fast food and drinking soft drinks more than three times a week and the likelihood of obesity, with notably high odds [36],[37]. The higher frequency of consuming fast food and soft drinks was consistently linked with being overweight and obese, aligning with findings from prior research. Moreover, a gender-specific trend emerged regarding the consumption of fast food and soft drinks and the prevalence of being overweight, indicating a significant relationship among boys but not girls. Additionally, the consumption of fast food and drinking soft drinks exhibited a noteworthy independent association with higher BMI [3]. Diet remained one of the most significant risk factors for being overweight and obese, making it the most common nutritional problem among school-aged children and adolescents [43]. Multiple studies have shown that the majority of children and adolescents did not meet the dietary guidelines for food intake [44]. Instead, they consumed excessive amounts of nutrient-poor, high-sugar, and high-fat foods while not consuming enough fruits and vegetables [45]. These dietary choices impacted their calorie intake, dietary patterns, and promoted habits such as an increased consumption of fast-food and sugar-sweetened beverages, which are associated with obesity and being overweight [42]. Positive nutrition behaviors are crucial for children and adolescents' growth, healthy weight, and short- and long-term health outcomes [46].

The current study significantly contributes to understanding health challenges in Pakistan and global health trends, particularly regarding nutrition behavior and body weight issues among school children and adolescents. One strength is its provision of actual data from Pakistan, offering insights into these correlations. Unlike previous studies that were limited to specific age groups or locations, our sampling technique ensured a broader representation. Additionally, it was the first to comprehensively explore this relationship in Pakistan. Despite some nonsignificant findings, our study underscores the need for nuanced analyses and informed interventions and policies to address obesity and being overweight. By focusing on an underrepresented population, our research fills critical gaps, providing updated insights into prevalence rates among Pakistani youth.

The current study encountered several major limitations. First, its cross-sectional design prohibits causal inference for the observed associations. Additionally, relying on self-reported data on diet-related behaviors introduces the potential for information bias. Moreover, solely focusing on school-age children and adolescents aged 9 to 17 years limits the generalizability of the findings to the broader Pakistani population. Excluding younger age groups (grades 1 through 3) may overlook crucial factors related to being overweight and obese during primary school years. Furthermore, some girls' school principals' reluctance to permit measurements for girls aged 12 to 17 years affected the representation of girls in the sample. Notably, obesity and being overweight were solely determined based on BMI calculations using the US CDC 2000 reference BMI chart, neglecting the assessment of body fat percentage, which could provide a more comprehensive measure of adiposity. These limitations highlight the need for the careful interpretation of our findings and suggest avenues for future research to address these gaps.

## 5. Conclusions

In conclusion, the study highlighted the concerning rise in the obesity prevalence and being overweight among Pakistani school-aged children and adolescents. The findings underscored a significant association between these conditions and the nutrition behavior/food intake. Encouraging regular breakfast consumption, frequent fruit and vegetable intake, and discouraging the consumption of junk foods and soft drinks could aid in maintaining healthier body weights. These recommendations align with guidelines from reputable health organizations such as the US CDC, the WHO, and Chinese and Canadian authorities. The implementation of such strategies should involve a comprehensive, multisectoral approach that targets children, adolescents, parents, teachers, and healthcare professionals across families, schools, and primary health settings. Special attention should be given to younger age groups and adolescents from lower socioeconomic backgrounds. Gender-specific differences in dietary behaviors warrant further investigations for more effective obesity prevention and control measures. Establishing supportive networks among parents, teachers, and school administrations can enhance the psychological environment for students, which can be complemented by psychological assistance initiatives both at school and within the home. Regular anthropometric surveys in schools, along with teacher training on growth chart utilization, are recommended for ongoing monitoring and intervention. Future research utilizing longitudinal or interventional designs can offer deeper insights into the relationship between being overweight, obesity, behavioral factors, and other health indicators, thereby informing more targeted interventions.

## Use of AI tools declaration

The authors declare they have not used Artificial Intelligence (AI) tools in the creation of this article.
